# FAM65B controls the proliferation of transformed and primary T cells

**DOI:** 10.18632/oncotarget.11438

**Published:** 2016-08-20

**Authors:** Jeanne Froehlich, Margaux Versapuech, Laura Megrelis, Quitterie Largeteau, Sylvain Meunier, Corinne Tanchot, Georges Bismuth, Jérôme Delon, Marianne Mangeney

**Affiliations:** ^1^ Inserm, Institut Cochin, Paris, France; ^2^ Cnrs, Paris, France; ^3^ Université Paris Descartes, Sorbonne Paris Cité, Paris, France; ^4^ Inserm, PARCC, Université Paris Descartes, Sorbonne Paris Cité, Paris, France

**Keywords:** cell proliferation, cell cycle, signaling, T lymphocytes, leukemia

## Abstract

Cell quiescence is controlled by regulated genome-encoded programs that actively express genes which are often down-regulated or inactivated in transformed cells. Among them is FoxO1, a transcription factor that imposes quiescence in several cell types, including T lymphocytes. In these cells, the FAM65B encoding gene is a major target of FOXO1. Here, we show that forced expression of FAM65B in transformed cells blocks their mitosis because of a defect of the mitotic spindle, leading to G2 cell cycle arrest and apoptosis. Upon cell proliferation arrest, FAM65B is engaged in a complex containing two proteins well known to be involved in cell proliferation i.e. the HDAC6 deacetylase and the 14.3.3 scaffolding protein. In primary T cells, FAM65B is down-regulated upon T cell receptor engagement, and maintaining its expression blocks their proliferation, establishing that the decrease of FAM65B expression is required for proliferation. Conversely, inhibiting FAM65B expression in naive T lymphocytes decreases their activation threshold. These results identify FAM65B as a potential new target for controlling proliferation of both transformed and normal cells.

## INTRODUCTION

Before encountering foreign antigens, naive T lymphocytes do not proliferate. It was initially assumed that this “default” state reflected the absence of activating signals [1]. It is now known that the steady-state maintenance of naive T cells is an active state and depends on a combination of signals from the IL-7 receptor and the T cell antigen receptor (TCR) triggered by major histocompatibility complex (MHC) molecules loaded with self-peptides [2, 3]. Paradoxically, the same signals lead to a loss of quiescence of naive T cells in some situations such as lymphopenia, and result in entry into the cell cycle (lymphopenia-induced proliferation) and acquisition of memory-like markers [2, 3]. Therefore, naive T cells are somehow restrained in proliferation, but this restraint can be alleviated by external cues. The molecular mechanisms underlying such restraint have proven elusive.

The forkhead box O1 (FoxO1) transcription factor is involved in the maintenance of the quiescence state of T cells [4]. The FoxO family is the mammalian orthologue of the *c-elegans* protein DAF16, and has an evolutionarily conserved function in the adaptation of proliferation-to-nutrient availability [5]. In quiescent T cells, FoxO1 is nuclear, and binds DNA. This DNA binding drives the transcription of several genes that encode proteins involved in cell mobility, cell survival and quiescence. Upon TCR stimulation, FOXO1 is phosphorylated by Akt kinase under the control of the phospho-inositide 3 kinase (PI3K) pathway, leading to its nuclear exclusion and an arrest of its transcriptional activity [6, 7]. Conditional deletion of Foxo1 in mouse T cells results in spontaneous activation of T cells with an activated-memory phenotype [8].

We previously identified family with sequence similarity 65, member B (FAM65B; previously known as C6ORF32, KIAA0386 or PL48), as a transcriptional target of FOXO1 in T cells [7, 9]. Two main isoforms of FAM65B proteins are expressed in T cells and have been functionally characterized as an atypical inhibitor of the small G protein RhoA [9, 10]. FAM65B has also been described to induce neurite-like outgrowths in HEK293 and C2C12 cells probably through an action on microtubules [11]. This step appears to be involved in myoblast fusion and differentiation [12]. More recently, the protein has been shown to be a component of hair cell stereocilia, an actin-rich structure required for hearing [13]. The FAM65B protein does not seem to be endowed with intrinsic enzymatic properties. Instead, its functional effect in cell mobility seems to rely on its interaction with the small G protein RhoA [9, 10], whereas its role in myoblast differentiation is dependent on its interaction with a complex containing the histone deacetylase HDAC6 and 14.3.3 proteins [10, 12]. The 14.3.3 proteins are a family of regulatory signaling molecules that interact with other proteins in a phosphorylation-dependent manner and function as adapter or scaffold proteins in signal transduction pathways [14]. Although 14.3.3 proteins act in cell signaling, cell cycle control, and apoptotic cell death, a large group of 14.3.3 -binding partners have been described to regulate cytoskeleton architecture [15].

We now report that FAM65B can act as a molecular switch controlling quiescence of normal T cells and proliferation of malignant cell lines. Analyzing the mechanism responsible for this effect, we show that proliferating cells are blocked in mitosis due to a defect of the mitotic spindle triggered by FAM65B overexpression. We also demonstrate at the molecular level that FAM65B forms a molecular complex with HDAC6 and 14.3.3, and that this tripartite complex is required for proliferation arrest. We also show that quiescent T lymphocytes express high levels of FAM65B and that a rapid down-regulation of the molecule is necessary to enable T cells to divide in response to TCR engagement. Accordingly, we also show that FAM65B cellular levels set the activation threshold of T cells required to start a substantial proliferation.

## RESULTS

### FAM65B inhibits the proliferation of human leukemic T cells

**Figure 1:**
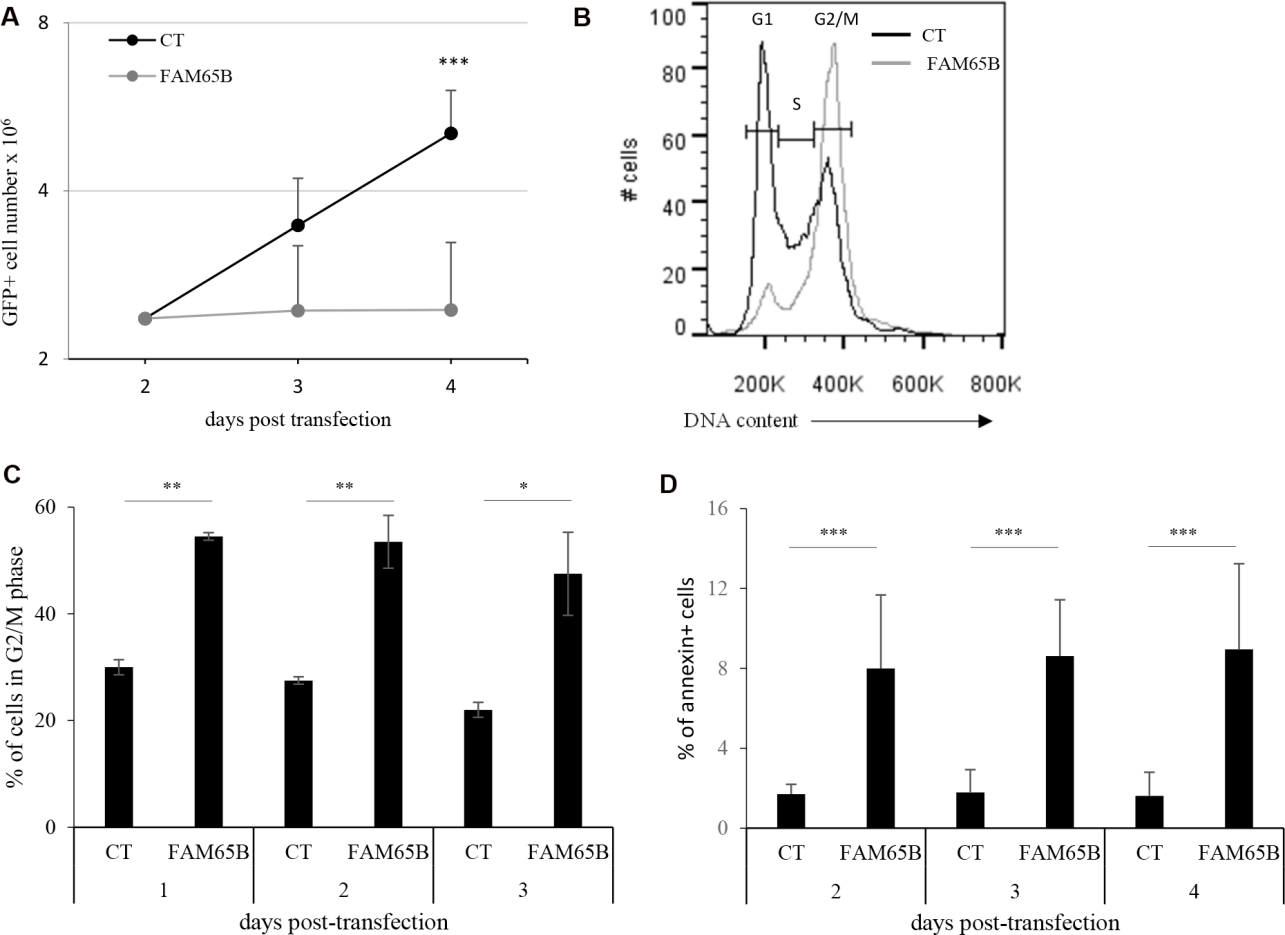
FAM65B expression inhibits cell proliferation by perturbing mitosis stage (**A**) Jurkat cells were transfected either with GFP or FAM65B fused with GFP expression vectors and their proliferation was measured by counting the total viable cell number at the indicated time points. (**B**) The cell cycle profile was evaluated by flow cytometry three days after transfection. (**C**) The proportion of transfected Jurkat cells in G2/M phase was estimated daily by flow cytometry. (**D**) The proportion of annexin V-labeled cells was measured daily by flow cytometry. Results are shown as means ± SD of at least three individual experiments. Statistical analysis: Paired two-tailed Student's *t*-test.

FAM65B is transcriptionally controlled by FOXO1 [9]. In the Jurkat leukemic T cell line, where the PI3K pathway is constitutively active, FOXO1 is permanently shut-down and so degraded [16] (Supplementary Figure S1A, lane 2), and the two isoforms of FAM65B are not expressed ([7, 9], Supplementary Figure S1B, lane 1). We therefore used these cells to follow how FAM65B re-expression could affect their growth. Cells were transfected with expression constructs coding for GFP alone as a control, or for FAM65B isoform 2 fused to GFP. Having confirmed that FAM65B re-expression did not alter FOXO1 expression level (Supplementary Figure S1A, lane 2 and 3), we monitored the proliferation by counting the total viable cell number daily, and quantifying the percentage of GFP^+^ cells by flow cytometry. In contrast to control cells, the number of FAM65B expressing cells did not increase over time (Figure 1A). The same effect was observed when FAM65B isoform 1 fused to GFP was expressed (data not shown). Analysis of the cell cycle demonstrated that FAM65B expression results in a G2/M accumulation after 3 days of culture, with 47 ± 7% (mean ± SD) of FAM65B positive *vs* 22 ± 1.4% of control cells in G2/M phase (Figure 1B and 1C). In addition, annexin V labelling revealed that the percentage of dying cells was significantly increased by FAM65B (Figure 1D).

### FAM65B is a mitotic inhibitor that alters the formation of the mitotic spindle

**Figure 2:**
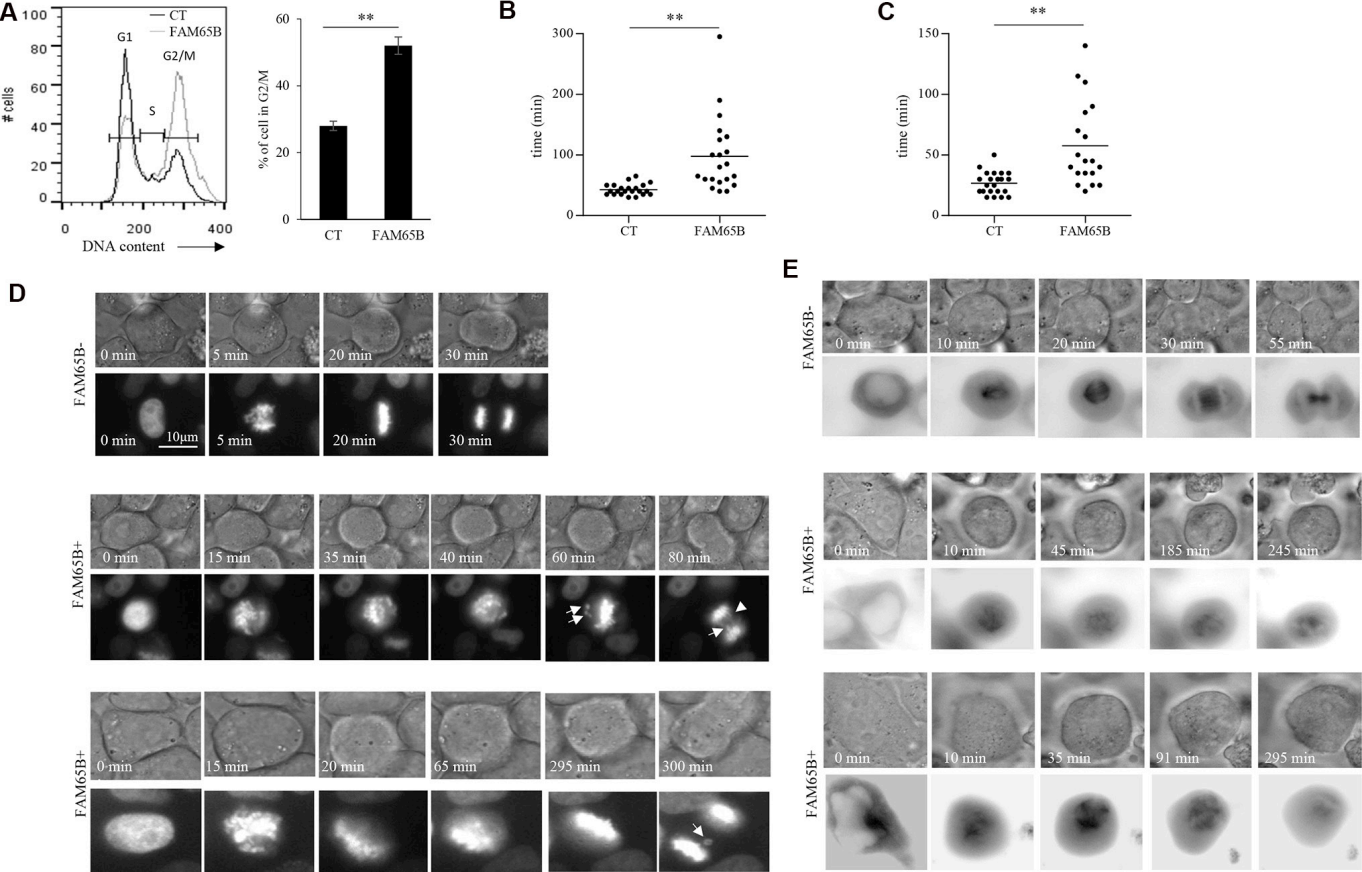
FAM65B expression disrupts the formation of the mitotic spindle, resulting in mitotic failure (**A**) The cell cycle of HeLa cells transfected with GFP alone or FAM65B fused with GFP expression vectors was analyzed by flow cytometry two days after transfection. Results are representative of three experiments. The time required for each HeLa cell co-transfected with Histone H2B-mCherry and GFP (*n* = 22) or FAM65B-GFP (*n* = 27) expressing vectors to complete a division (**B** and **D**) or to align chromosomes (**C**) was evaluated by time-lapse microscopy. Statistical analysis: Paired two-tailed Student's *t*-test. (**E**) HeLa cells were co-transfected with α-tubulin-GFP and m-Cherry (*n* = 14) or FAM65B-mCherry (*n* = 20), and their capacity to form a mitotic spindle was followed by time-lapse microscopy.

In order to study in detail the mechanism of this FAM65B-induced growth arrest, we used the classic model of adherent HeLa cancer cell line. HeLa cells, as all cell lines that we have tested including HEK293, NIH3T3 and CEM cells, do not express FAM65B (data not shown). We first show that, as observed in Jurkat cells, FAM65B expression led to an increase of HeLa cells in G2/M phase (55% vs 29% in control cells; Figure 2A). Next, in order to analyze how FAM65B might disturb the mitotic process, we co-transfected HeLa cells with vectors coding for GFP alone or FAM65B-GFP, and histone H2B-mCherry, to monitor the dynamics of DNA reorganization during mitosis. Whereas cells transfected with GFP alone completed mitosis in 43 ± 9 min, the time required for mitosis of FAM65B-expressing cells was more variable and longer (94 ± 65 min, Figure 2B and 2D). Chromosomes in the control cell were perfectly aligned on the metaphase plate 16 ± 5 min after initiation of cell division, as determined by cell rounding (Figure 2C and 2D). In contrast, FAM65B-expressing cells were not able to align chromosomes correctly, leading to abnormal anaphases with frequent lagging chromatids after more than 20 min for 37% of the cells, and more than 40 min for 14% of the cells (Figure 2C and 2D).

Chromosome movements during mitosis rely upon a complex macromolecular machinery known as the mitotic spindle. The spindle has three principal components, centrosomes, microtubules composed of α-tubulin, and chromosomes [17]. Since FAM65B is described as regulating the elongation of microtubules [18], we supposed that FAM65B expression might perturb the formation of the mitotic spindle. To test this hypothesis, we co-transfected HeLa cells with vectors coding for GFP alone or FAM65B-GFP, and α-tubulin-mCherry, to follow the microtubule network during mitosis. We observed that, whereas cells transfected with GFP alone were able to form a mitotic spindle, FAM65B-expressing cells presented an aberrant tubulin network with an absence of clear mitotic spindle (Figure 2E). These results show that FAM65B expression disturbs the formation of the mitotic spindle, resulting in mitotic failure.

### The FAM65B/HDAC6/14.3.3 tripartite complex is required for proliferation arrest

**Figure 3:**
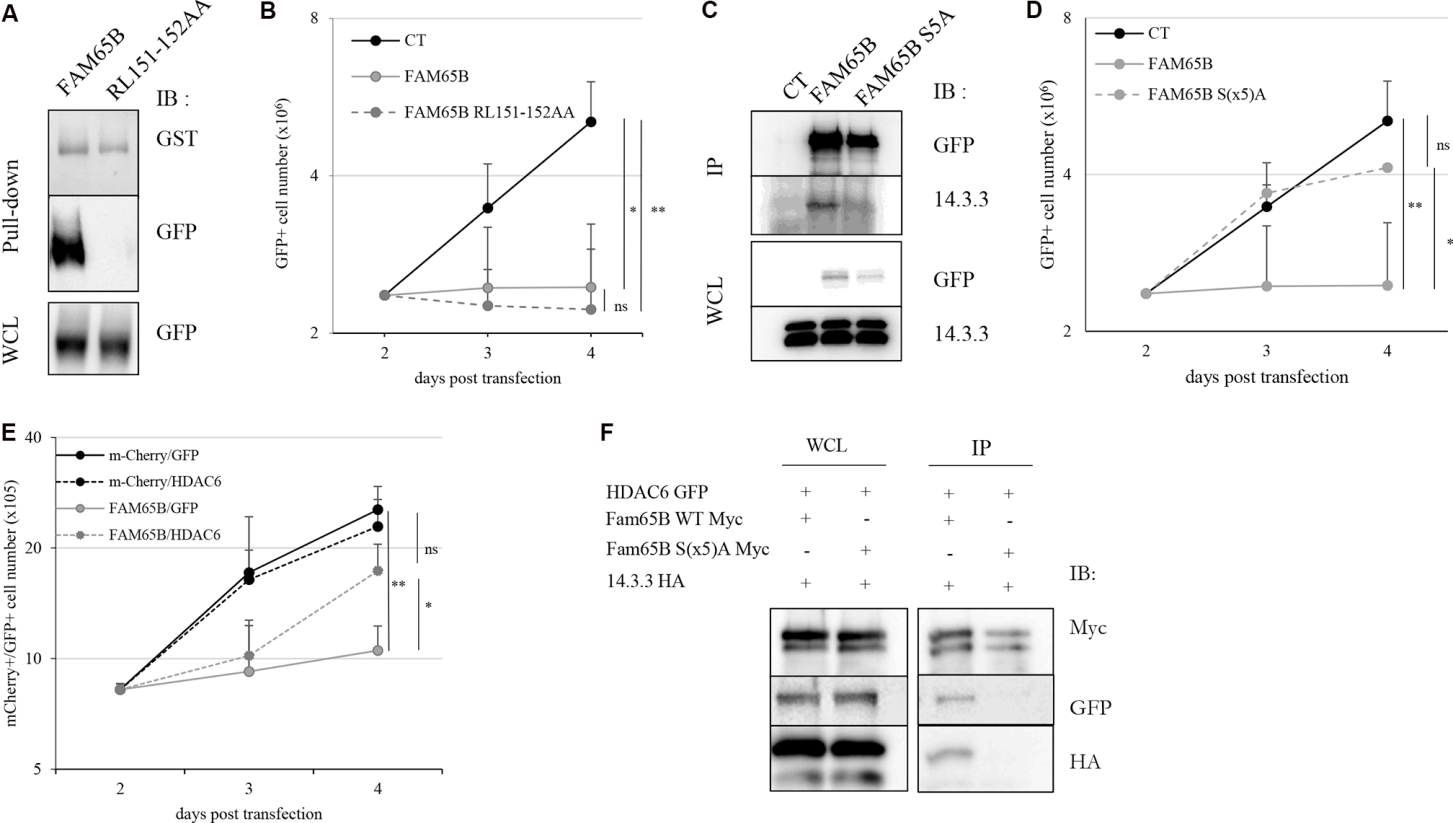
The anti-proliferative property of FAM65B results from its capacity to interfere with HDAC6 and 14.3.3 (**A**) Lysates from HEK293T cells transfected with FAM65B-GFP or FAM65B RL151-152AA-GFP mutant were subjected to a pull-down assay using beads bearing GST-RhoA. (**B**) The proliferation of transfected Jurkat cells with GFP alone (Control: CT) and wild type or mutant RL151-152AA forms of FAM65B expression vectors was measured by daily counting the number of viable cells. Results are shown as means ± SD of at least three individual experiments. (**C**) HEK293T cells were transfected with pEF-Myc vector expressing either no protein (Control: CT), FAM65B or its mutant S(x5)A form fused to GFP. Whole cell lysates (WCL) and anti-Myc immunoprecipitates were analyzed by immunoblotting. (**D**) The proliferation of transfected Jurkat cells with either GFP alone (Control: CT), wild type or mutant S(x5)A of FAM65B expression vectors was measured by counting daily the number of viable cells. Results are shown as means ± SD of at least three individual experiments. (**E**) The proliferation of Jurkat cells co-transfected with mCherry or FAM65B-mCherry and GFP or HDAC6-GFP was measured by counting the number of viable cells every day. Results are shown as means ± SD of at least three individual experiments. (**F**) HEK293T cells were co-transfected with HDAC6-GFP, 14.3.3-HA and pEF-Myc vector expressing either FAM65B or its mutant S(x5)A form. Whole cell lysates and anti-Myc immunoprecipitates were analyzed by immunoblotting.

We then examined the implication of known targets of FAM65B in the molecular mechanism of growth arrest. We have previously shown that FAM65B binds directly and inhibits RhoA [9], a protein that has been involved in cytokinesis [19]. We hypothesized that the effect of FAM65B on proliferation could be due to this interaction. Therefore, we constructed a plasmid coding for a mutant version of FAM65B unable to bind RhoA (RL151-152AA, [10] and Figure 3A). Upon transfection, this mutant was still able to inhibit proliferation of Jurkat T cells as efficiently as the wild type FAM65B protein, thus showing that the anti-proliferative activity of FAM65B was not due to its RhoA inhibitory activity (Figure 3B).

It has been shown that the phosphorylated form of FAM65B can interact with 14.3.3, a family of proteins that bind to phospho-Serine and phospho-Threonine residues and play important roles in various processes, including in coordinating cell cycle progression [14]. As FAM65B has been described as a phosphorylated protein in neutrophils [10], we examined its phosphorylation status in Jurkat T cells by treating whole cell extracts with λ-phosphatase. We observed a decrease of the apparent molecular weight of the protein (Supplementary Figure S1B), suggesting that FAM65B is also phosphorylated in these cells. A previous report has shown that a mutant form of FAM65B with serine residues mutated to alanine at positions 21, 37, 341, 523, and 535, (hereafter called FAM65B S(x5)A) did not bind 14.3.3 anymore [10], see also Figure 3C). We therefore investigated whether this mutant could have an anti-proliferative effect. We observed that the S(x5)A phosphorylation mutant of FAM65B lost its capacity to inhibit the proliferation of Jurkat cells (Figure 3D). Therefore, the phosphorylation of FAM65B and its interaction with 14.3.3 is required for its anti-proliferative activity.

It has been stated that (i) 14.3.3 is a molecular partner of HDAC6 [20], (ii) inhibition of the deacetylase activity of HDAC6 leads to inhibition of cell proliferation, and (iii) FAM65B inhibits the deacetylase activity of HDAC6 [12]. We therefore speculated that the anti-proliferative effect of FAM65B might rely on its capacity to inhibit HDAC6 through its interaction with 14.3.3. To test this hypothesis, we first tried to reverse the anti-proliferative effect of FAM65B by an excess of HDAC6 in FAM65B-arrested Jurkat cells. The results showed that an excess of HDAC6 was able to partially override the FAM65B-induced proliferation arrest (Figure 3E). Conversely, inhibition of HDAC6 with a pharmacological inhibitor dose-dependently inhibited the growth of Jurkat cells and induced a marked G2/M arrest, mimicking the effect of FAM65B (Supplementary Figure S2A), in a similar fashion to what is normally observed with etoposide (Supplementary Figure S3). We next assessed the interaction between FAM65B, 14.3.3, and HDAC6, and the role of FAM65B phosphorylation in these interactions. To do this, we co-transfected HDAC6 and 14.3.3 with wild type or S(x5)A form of FAM65B. Whereas HDAC6 and 14.3.3 co-immunoprecipitate with the wild-type form of FAM65B (Figure 3F, lane 1), the mutated form of FAM65B was unable to interact with these two partners (Figure 3F, lane 2). These results suggest that the anti-proliferative property of FAM65B results from the capacity of its phosphorylated form to interact with HDAC6 and 14.3.3.

### FAM65B controls T cell proliferation upon TCR stimulation

**Figure 4:**
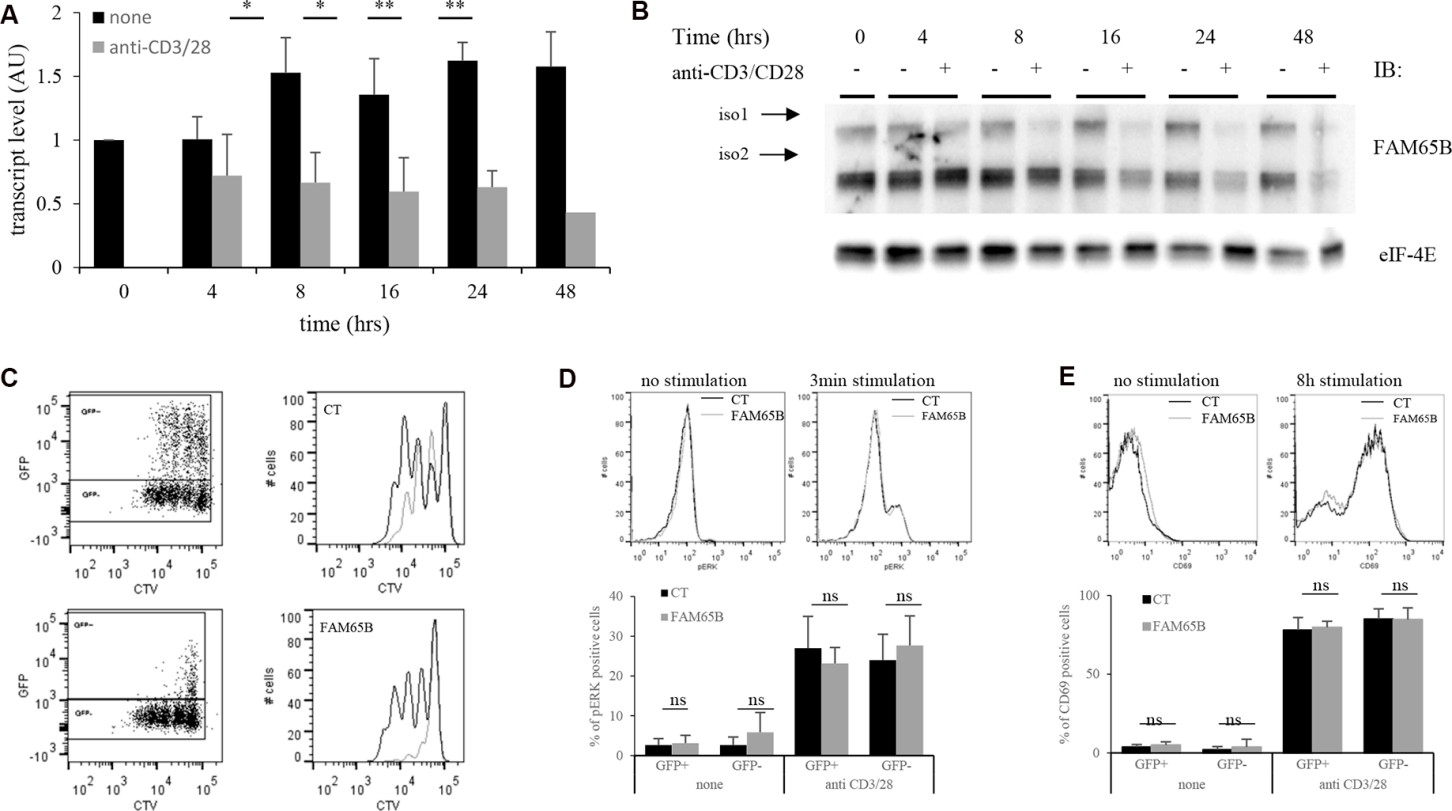
FAM65B expression is inversely correlated with the proliferative potential of activated T cells Primary human T cells were stimulated *in vitro* for several hours using anti-CD3 and -CD28 coated-beads. FAM65B expression was quantified at both the transcript (**A**) and the protein (**B**) levels. (**C**) Primary human T cells were transfected with GFP (Control: CT) or FAM65B-GFP expression vector then stained with Cell Trace Violet (CTV) and stimulated *in vitro* as described in (A) for 4 days. In the histograms, the grey lines correspond to the GFP^+^ cells and the black lines to the GFP^−^ cells. Note that in CT conditions, GFP^+^ cells proliferate slightly less efficiently than GFP^−^ cells. This is likely to be an effect of the transfection *per se*. (**D**) ERK phosphorylation and (**E**) CD69 expression of transfected cells stimulated or not with anti-CD3 and –CD28 was analyzed by flow cytometry after 3 minutes and 8 hours of culture, respectively.

**Figure 5:**
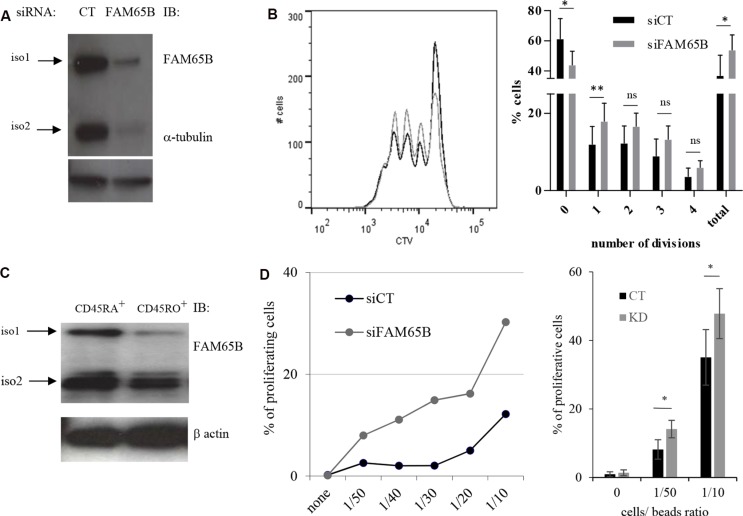
FAM65B expression is inversely correlated with the T cells activation threshold (**A**) The efficiency of FAM65B knock-down in human primary T cells was checked by immunoblotting with anti-FAM65B and anti-α-tubulin as a loading control. (**B**) Human primary T cells were transfected with control (siCT) or FAM65B (siFAM65B) siRNA, then stained with CTV and stimulated *in vitro* for 3 days using anti-CD3 and -CD28 coated-beads. The proliferation of transfected cells was analyzed by flow cytometry after 3 days of stimulation. The percentage of cells exhibiting 0, 1, 2, 3, 4 divisions or the addition of them (total) is quantified as the mean ± SD obtained from four donors. (**C**) Naive (CD45RA+) or activated/memory (CD45RO+) populations were purified from peripheral blood T cells. FAM65B expression was then analyzed by immunoblotting. (**D**) Primary human CD45RA^+^ T cells transfected with siFAM65B or siCT were stained with CTV and stimulated *in vitro* using variable doses of anti-CD3 and anti-CD28 -coated beads. The proliferation of the transfected cells was analyzed by flow cytometry after 3 days of stimulation. Results are representative of at least three individual experiments

We next analysed the consequences of the regulation of cell division by FAM65B upon physiological T cell activation. We first followed the expression level of FAM65B in T cells upon TCR triggering to see whether it was subject to variations after stimulation. FAM65B expression was quantified at the transcript and protein levels, following *in vitro* stimulation of primary human T lymphocytes with anti-CD3 and -CD28 coated beads. FAM65B transcripts decreased quickly upon TCR stimulation (Figure 4A), in line with a general and early block in FOXO1-mediated transcription upon antigen recognition [6]. Both FAM65B isoforms show a simultaneous loss, which was complete 24 hours after the onset of stimulation (Figure 4B). This observation is not related to cell culture as the level of FAM65B is constant in non-stimulated cells. To reveal the functional importance of this decrease, we next followed the consequences of maintaining FAM65B expression during activation. Primary human T cells were transfected with a plasmid coding for FAM65B-GFP (or GFP alone as a control), activated with anti-CD3 and -CD28 coated beads, and cell proliferation was monitored by analyzing cell trace violet (CTV) dilution of GFP^+^ cells. We observed that expression of FAM65B totally inhibits the proliferation induced by TCR engagement (Figure 4C). ERK phosphorylation (Figure 4D) and CD69 expression (Figure 4E) upon TCR stimulation was similarly induced in both conditions, excluding any major defect in early TCR signaling upon FAM65B expression. Again, we also observed that addition of an HDAC6 inhibitor on primary T cells stimulated with anti-CD3 and -CD28 coated-beads has the same effects than FAM65B, leading to an inhibition of proliferation with no cell death (Supplementary Figure S2B). With a complementary approach, we used RNA interference (RNAi) to study the consequences of decreasing FAM65B expression prior to TCR stimulation of primary human peripheral blood T cells (Figure 5A). We observed that FAM65B-depleted cells proliferated more than control cells (Figure 5B).

Primary peripheral T cells have a heterogeneous capacity to proliferate based on CD45RA expression ([21] and Supplementary Figure S4). We purified naive CD45RA+ (high activation threshold) and CD45RO+ (low activation threshold) populations from peripheral blood T cells, and sought to determine whether the lower activation threshold of CD45RO+ T cells was associated with decreased FAM65B expression levels. Indeed, our results show that these cells express a lower level of FAM65B than naive CD45RA+ cells (Figure 5C).

We then studied the proliferation of CD45RA+ cells upon activation with increasing doses of anti-CD3 and -CD28 coated-beads after FAM65B depletion. For all experiments, the depletion of FAM65B was verified by intracellular staining and flow cytometry analysis (Supplementary Figure S5). In the absence of stimulation, no proliferation was observed. However, FAM65B-depleted cells were induced to proliferate at sub-optimal doses of activators that induced only marginal proliferation of control T lymphocytes expressing FAM65B (Figure 5D). These results show that there is an inverse correlation between the cellular levels of FAM65B and the capacity of human primary T cells to proliferate in response to an activation signal. They ultimately suggest that the decrease of FAM65B expression is a pre-requisite for T-cell proliferation.

## DISCUSSION

We show here that FAM65B, a major transcriptional target of FOXO1, known as a quiescence factor, inhibits the proliferation of transformed cell lines through perturbation of mitosis. Our data suggest that this effect is linked to the capacity of FAM65B to inhibit the HDAC6/14.3.3 cell proliferation control pathway. In primary T cells, we observed that FAM65B expression regulates cell proliferation. Finally, our results indicate that the level of expression of FAM65B might also set the activation threshold of sub-populations of T cells.

Our work establishes that inhibition of FAM65B expression is required for cell proliferation as its ectopic expression in proliferating cells induces cell cycle arrest and death. Therefore, FAM65B could be considered as a *bona fide* quiescence factor. It was initially thought that cells spontaneously become quiescent. It is now believed that quiescence is a cellular state controlled by environmental cues and required for tissue physiology, as demonstrated for instance in the hematopoietic system [22]. As a consequence, much recent work has focused on the active regulation of this state [23]. Although the link between loss of quiescence and proliferation is intuitive, some observations obtained in the T cell model are intriguing. Indeed, one notable feature observed upon TCR stimulation is a comparatively long time period (> 24 h) before the first cell division, as compared with the subsequent cell divisions that involve rapid doubling (every 5–6 hrs) [24]. The state through which naive T cells transit, from quiescence to the entry in the first cell cycle, is not fully characterized even if some metabolic events have been involved [25]. FAM65B was initially reported to be expressed in syncytia, both in placenta and in muscle [11, 18, 26]. In syncytia, multinucleated cells result from cell-cell fusion induced by endogenous retroviral envelops [27, 28]. Interestingly, it has recently been established that the cell-cell fusion induced by viruses led to cell cycle arrest and senescence, i.e. irreversible quiescence [29]. In addition, terminal differentiation of HL-60 cells led to both cell cycle arrest and FAM65B expression [26]. We now report that FAM65B is highly expressed in naive T lymphocytes, a cell population that is characterized by a reversible quiescent state, and is down-regulated upon cell cycle entry. The expression of FAM65B can therefore be considered as a molecular marker of quiescence, whether or not it can be reversed.

FoxO1 is known to control cell proliferation through multiple mechanisms. FoxO factors stabilize G1 and G2/M checkpoints by the activation of a number of cell cycle regulatory proteins, including p130, members of the Cip/Kip and INK4 CKI families, cyclin G and Gadd45a. In addition, FoxO factors can also facilitate cell cycle arrest through the inhibition of the D-type cyclins and FoxM, a subgroup of Fox transcription factors that is known to positively drive G2/M phase transition [30]. In this study, we show that FAM65B, a transcriptional target of FOXO1, induces G2/M arrest. As previously proposed [12, 18], one mechanism to explain this effect might be an inhibition of the microtubule network by FAM65B, and especially a regulation of tubulin acetylation by FAM65B. Indeed, we know that FAM65B inhibits the deacetylase activity of HDAC6, a histone deacetylase of class II that functions as a tubulin deacetylase [12]. This hypothesis about a role of HDAC6 in the effects of FAM65B is further validated by our results showing that the cell cycle arrest induced by FAM65B can be overcome by an excess of HDAC6. It should also be noted that pharmacological inhibitors of HDAC6, which are used in clinical trials for the treatment of myeloma [31–33], mimic the anti-mitotic effect of FAM65B. In previous studies, HDAC6 activity was established as a requirement for efficient oncogenic transformation. Without HDAC6 expression, fibroblasts were more resistant to both Ras- and ErbB2-dependent transformations [34]. The absence of FAM65B expression in transformed cell lines, consecutive to the inhibition of FOXO1 transcriptional activity, might also participate in the deregulation of HDAC6 required for the transformation process. Thus, as observed in our experiments with transformed cells, the perturbed mitosis induced by FAM65B could be the result of a defective microtubule organization downstream of HDAC6, culminating in a failure of the mitotic spindle during mitosis. Interestingly, FAM65B was recently localized to the plasma membrane of the stereocilia of both inner and outer hair cells, and mutations of FAM65B leads to deafness syndromes, suggesting that the lack of functional FAM65B could hinder the development of the mechanotransduction apparatus, through perturbation of the primary cilium [13]. Indeed, HDAC6 has been notably implicated in cell proliferation *via* the regulation of the primary cilium [35, 36], which is known to control the cell cycle [37].

In addition, our work shows that the phosphorylation of FAM65B is required for both this anti-proliferative function and the formation of a tripartite complex comprised of FAM65B, 14.3.3 and HDAC6. Several studies have identified a possible phosphorylation of FAM65B not only in the Jurkat leukemic T cell line [38], but also in primary mouse neutrophils [10] and T lymphocytes [39]. In this latter case, the phosphorylation results from PKCη activity [39]. The 14.3.3 scaffolding proteins play a critical role in the regulation of cell fate through phospho-dependent binding to a large number of intracellular proteins. This function can be achieved by the formation of multi-protein complexes upon recruitment of such proteins or by restricting the access of other ones [14]. FAM65B, as a well-known 14.3.3 phosphopeptide ligand, contains the consensus sequences RSXpT/SXP and RXY/FXpT/SXP. The study by Gao *et al*. [10], confirmed by our results, show that the phosphorylation of FAM65B allows its interaction with 14.3.3. Though it cannot be excluded that phosphorylated form of FAM65B directly interacts with HDAC6, the fact that the FAM65B/HDAC6 interaction has been observed only in the context where the three proteins were present and not detected in our yeast two-hybrid screen [9], suggests that 14.3.3 makes a link between HDAC6 and FAM65B.

One intriguing feature of our work is the regulation of the T cell activation threshold by FAM65B. In T lymphocytes, the strength and duration of TCR signaling in response to antigen stimulation dictates the magnitude and the quality of the primary response [40, 41]. The TCR activation threshold, agonist affinity discrimination as well as signal termination must therefore be tightly regulated. This is achieved through the concerted action of multiple positive and negative regulators acting in resting or activated T cells. The 14.3.3 scaffolding protein has already been involved in the fine tuning of TCR-activation through relocalization of several signaling proteins [42–45], including FoxO1 [46, 47], suggesting a possible amplification loop that would keep FoxO1 in the cytosol and therefore would maintain FAM65B expression levels very low in activated T cells. Although much work will have to be done to precisely understand how FAM65B is able to set the activation threshold of T cells, it is tempting to assume, on the basis of our results, that its capacity to control the activity of the 14.3.3/HDAC6 complex could participate in this function.

We have already shown that in T lymphocytes, FAM65B inhibits cell migration by blocking the GTP loading of RhoA [9]. Here, we show that FAM65B has a general quiescence promoting activity, which is active in resting T cells and controls both proliferation and setting of the activation threshold, at least in part through the 14.3.3/HDAC6 pathway. Therefore, in T cells, FAM65B appears as a main effector target of FOXO1 that is able to interact with two different targets to control the two main cellular processes of activation, i.e. migration and proliferation.

## MATERIALS AND METHODS

### Cells

Human T lymphocytes were purified from the blood of healthy donors (Etablissement Français du Sang, Paris, France; relevant ethical approvals and consent have been obtained for use of human samples, ref E-2075) and cultivated as described [7, 9]. The naive (CD45RA+) and memory (CD45RO+) T cells were obtained by negative depletion on magnetic beads [Anti Human CD45RO Particles, Anti Human CD45RA Particles, respectively (BD Biosciences). Jurkat cells (gift of G. Crabtree, Stanford University, Stanford, CA) were cultivated in complete RPMI medium, HeLa (gift of C. Berlioz, Institut Cochin, France) and HEK293T cells (ATCC-CRL-3216) were cultivated in complete DMEM medium.

### Constructs and pharmacological inhibitors

pEGFP-FAM65B plasmids were previously described [9]. Mutations of the RhoA interaction domain (RL151-152AA) and phosphorylation sites (S(x5)A) were constructed by PCR using QuickChange II site-directed mutagenesis Kit (Agilent) with the primers listed in Supplementary Table S1. pmCherry-FAM65B was constructed by PCR by introducing the mCherry ORF at the *Age*I/*Not*I site of pEGFP-FAM65B. FAM65Bwt and S(x5)A Myc constructs were obtained by amplification with primers listed in Supplementary Table S1 and by introducing FAM65B ORF in pEF vector at the *Spe*I/*EcoR*V sites. plncx-Histone 2B-mCherry and plncx2-Tubuline-YFP were a kind gift of C. Berlioz (Institut Cochin, France) and pEGFP-N1-HDAC6 was purchased from Addgene (# 36188). HA-tagged 14.3.3 plasmid was a kind gift of JL Andersen (Brigham Young University, Provo, UT). HDAC6 specific antagonist CAY-10603 (Selleckchem) has been described and characterized before [47]. Etoposide is a kind gift of R. Donné (Institut Cochin, Paris). λ Protein Phosphatase was from Millipore.

### Quantitative RT-PCR

Total RNA was prepared using an RNeasy mini kit (QIAGEN). cDNA was produced with the Advantage RT-for-PCR kit (Clontech Laboratories) using 1 μg total RNA and random hexamer priming in a final volume of 20 μL. Real-time quantitative PCR was performed using the LightCycler FastStart DNA Master plus SYBR Green kit (Roche Diagnostics). Genes of interest were detected using primers that had been designed with Oligo6 software (Molecular Biology Insights) and optimized to generate a single amplicon of 80–130 nucleotides. The sequences of the primers used in qRT-PCR experiments are in Supplementary Table S1.

### DNA and RNA interference transfections

HeLa and HEK293T cells were transfected with Lipofectamine 2000 (Invitrogen), according to the manufacturer's instructions. Jurkat cells (5 × 10^6^) in 500 μL of RPMI without phenol red were electroporated with 5 μg DNA using the BioRad system (270 V, 950 μF). Primary T cells (5 × 10^6^) were nucleofected using the Amaxa system (Lonza; kit T, U-014 program) with 5 μg DNA or 2 μL of a 100 μM solution of OTP SMARTpool RNAi (Dharmacon) directed against human FAM65B or non-targeting sequences as a control.

### Flow cytometry

Apoptosis was determined by staining with Annexin V-PE (BD Pharmingen, PE Annexin V Apoptosis Detection kit I) according to the manufacturer's instructions.

Proliferation of primary T cells was assessed by dilution of Cell Trace Violet kit (Invitrogen). After two washes in PBS, cells were resuspended at 10^6^ cells per mL in a 5 μM Cell Trace Violet solution. Cells were incubated in dark at 37°C for 20 min. After the loading, cells were washed with a volume of cold PBS 10% FCS corresponding to 5 times the loading volume. Cell suspension was collected and dispensed into 96-well round-bottom microtiter plates (Falcon; 2 × 10^5^ cells per well) and stimulated using anti-CD3/CD28 coated-beads (Dynabeads, Life technologies) for 3 days.

For cell cycle analysis of transfected cells (Jurkat and HeLa cells), 10^6^ cells were fixed with 4% paraformaldehyde for 10 min, permeabilized with 0,1% saponin, and incubated with PBS 1 mM EDTA Supplementary emented with 50 μg/mL of Propidium Iodide (Sigma) and 200 μg/mL of RNAse A (Sigma) for 30 min at 37°C.

For analysis of ERK phosphorylation in transfected PBT, 10^6^ cells were fixed with 4% paraformaldehyde for 10 min, permeabilized with methanol at −20°C for 1 hour, and incubated with anti-ERK^P^ antibody (# 4370, Cell Signaling Technology) in PBS 0.2% BSA for 30 min at room temperature and then with F(ab')2-*Goat anti-Rabbit* IgG (H+L) secondary antibody (#A-21246, Thermofisher). Cytometry data were obtained using a BD-LSR Fortessa flow cytometer (BD Bioscience). Data files were analyzed using FlowJo software (TreeStar).

### Fluorescence microscopy

Time-lapse microscopy was performed on HeLa cells grown on coverslips in a Zeiss Axiovert 200 microscope at 37°C with a 20× objective (LD Plan Neofluar). The camera CoolSnap HQ (Photometrics) was used to acquire images. Images were processed using ImageJ software.

### Biochemistry

Primary T cells were stimulated using anti-CD3/CD28 -coated beads (1 bead per 5 cells, Dynabeads, Life technologies) into 96-well round-bottom microtiter plates (Falcon; 2 × 10^5^ cells per well). Collected cells were lysed in lysis buffer [20 mM Bicine, pH7.6; 0.6% CHAPS complete protease and phosphatase inhibitor cocktail (Roche)] for 30 min on ice. Lysates were collected after centrifugation at 14,000 rpm for 10 min. Protein concentration in supernatants was quantified with the Bradford assay. SDS loading buffer was added to each sample and then boiled at 100°C for 5 min. An equal amount of proteins was loaded on 10% SDS-PAGE, transferred to nitrocellulose membranes, and immunoblotted with anti-FAM65B Ab (Abnova), anti-β-actin Ab (Sigma), anti-α-tubulin (Santa Cruz) or anti-eIF-4E (Santa Cruz) followed by a secondary HRP-labeled Ab (Jackson ImmunoResearch). Membranes were revealed by ECL (GE Healthcare Life Sciences) using a camera (Fusion FX7, VilberLourmat). For immunoprecipitation experiments, transfected cells were washed in cold PBS and lysed in 1 mL lysis buffer [10 mM HEPES pH 7.5; 150 mM KCl; 0.1%NP-40; 10 μM SAHA (Sigma Aldrich); 40 μM Tubacin (Sigma Aldrich); 50 μM Nicotinamide (Sigma Aldrich); complete protease and phosphatase inhibitor cocktails (Roche)] for 30 min in ice. Lysates were collected after centrifugation at 14,000 rpm for 10 min. 40 μL of each lysate was saved and used as a loading control. 20 μL of EZ myc beads (Sigma Aldrich) pre-washed 3 times with lysis buffer were added in the lysate and rotated for 2 h at 4°C. After rotating, the beads were washed 3 times with lysis buffer and resuspended into 100 μL of TBS1X containing 10 μg/mL of myc peptide for 30 min at 4°C. 70 μL of the supernatant containing the protein complexes without beads/Ab was taken off. Each sample was boiled at 100°C for 5 min after adding 5× Laemmli buffer, subjected to 12% SDS-PAGE, transferred to nitrocellulose membrane and immunoblotted with the indicated antibodies. Pull-down assays with beads containing GST-tagged RhoA (Cytoskeleton) were described in Rougerie *et al.* [9].

### Statistical analysis

Statistical significance was analyzed by paired two-tailed Student's *t*-test and expressed as a *p* value. The number of analyzed samples (n) is indicated. All experiments were performed at least as three independent experiments. When indicated, standard deviations are represented as scale bars on graphs. We used the following conventions to illustrate the *p* value: *0.01 < *p* < 0.05, **0.01 < *p* < 0.001, ***p < 0.001. ns: not significant.

## SUPPLEMENTARY MATERIALS FIGURES AND TABLES


