# Supplementary material for: FAM65B controls the proliferation of transformed and primary T cells
Source: Oncotarget. 2016 Aug 20;7(39):63215–25. doi: 10.18632/oncotarget.11438 (PMC5325358; doi:10.18632/oncotarget.11438)
Supplement: Supplementary file 1 [file oncotarget-07-63215-s001.pdf]

FAM65B controls the proliferation of transformed and primary T cells

Supplementary Materials

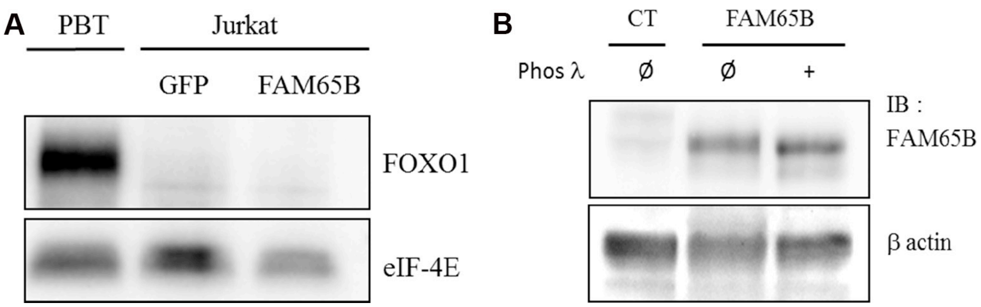

Supplementary Figure S1: Re-expression of FAM65B does not modify FOXO1 expression in Jurkat cell line and FAM65B is phosphorylated in Jurkat cell line. (**A**) Expression of FOXO1 analysis in lysates from peripheral blood T cells (PBT), used as positive control and Jurkat cells transfected with GFP or FAM65B GFP. (**B**) Lysates from Jurkat cells transfected with GFP or FAM65B GFP were treated with λ phosphatase and analyzed by immunoblotting with the indicated antibodies.

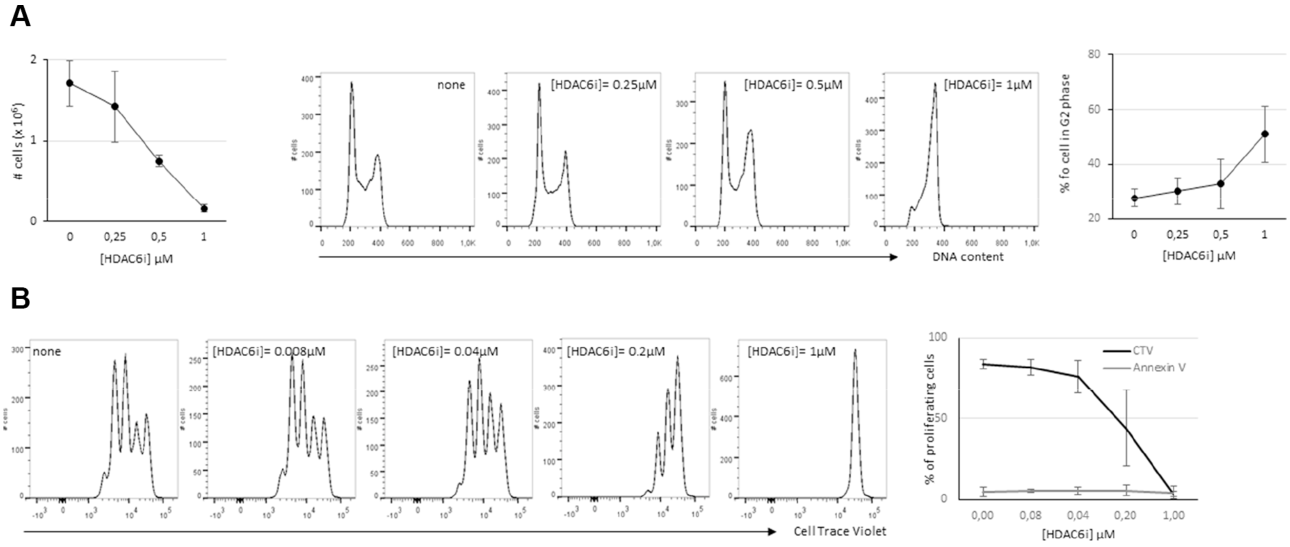

Supplementary Figure S2: Inhibition of HDAC6 inhibits T cell proliferation and blocks cell cycle progression in G2/M. (**A**) Jurkat cells were treated with increasing dose of HDAC6 inhibitor for 18 hours and their cell cycle was analyzed by flow cytometry. Results are representative of three experiments. Graphs represent the proliferation of cells (left panels) and the proportion of cells in G2/M phase (right panel) in function of the treatment. (**B**) Human primary T cells were stained with CTV and stimulated in vitro with anti-CD3 and anti-CD28 coated beads in presence of increasing dose of HDAC6 inhibitor. The effect of this inhibitor on cell proliferation and cell mortality was measured by flow cytometry after three days. Results are representative of three experiments. Means of the percentage of proliferating cells were plotted (right panel).

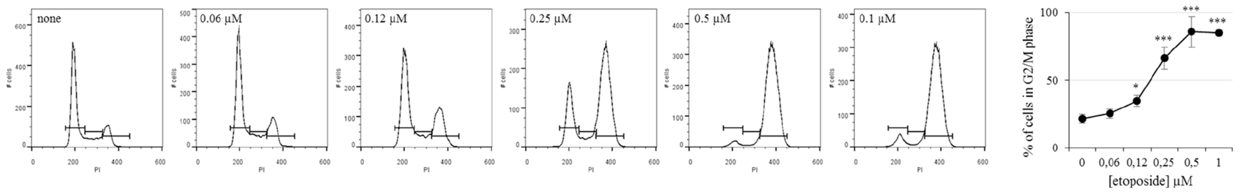

Supplementary Figure S3: Etoposide blocks cell cycle progression in G2/M. Jurkat cells were treated with increasing dose of etoposide for 18 hours and their cell cycle was analyzed by flow cytometry. Results are representative of three experiments. Graphs represent the proportion of cells in G2/M phase (right panel) in function of the treatment.

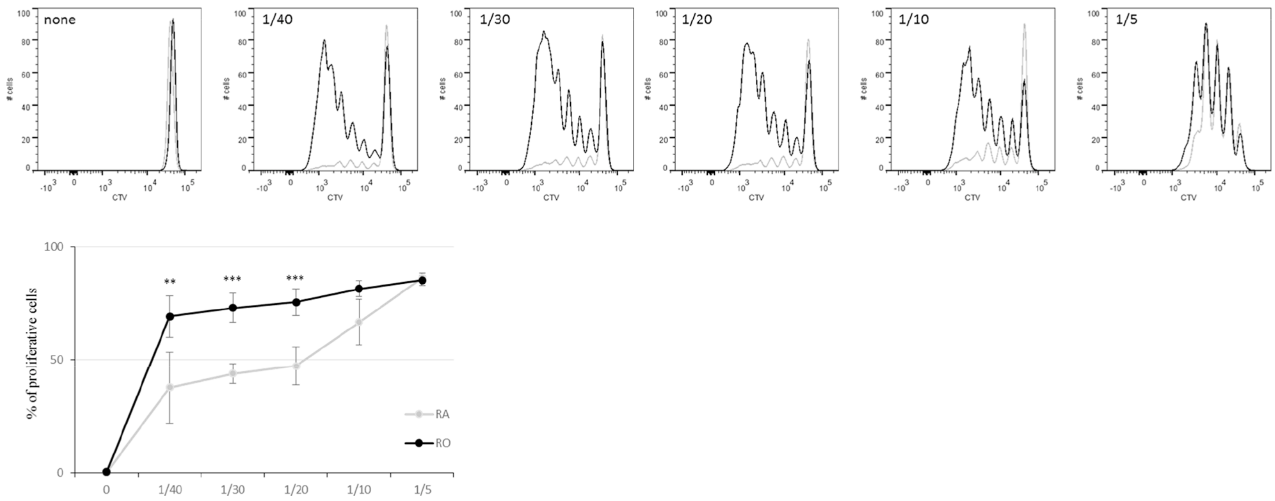

Supplementary Figure S4: CD45 RA positive cells are less prone to proliferate. Human primary CD45 RA+ and RO+ T cells were stained with CTV and stimulated in vitro with anti-CD3 and anti-CD28 coated beads. Cell proliferation was measured by flow cytometry after three days. Results are representative of three experiments. Means of the percentage of proliferating cells were plotted (lower panel).

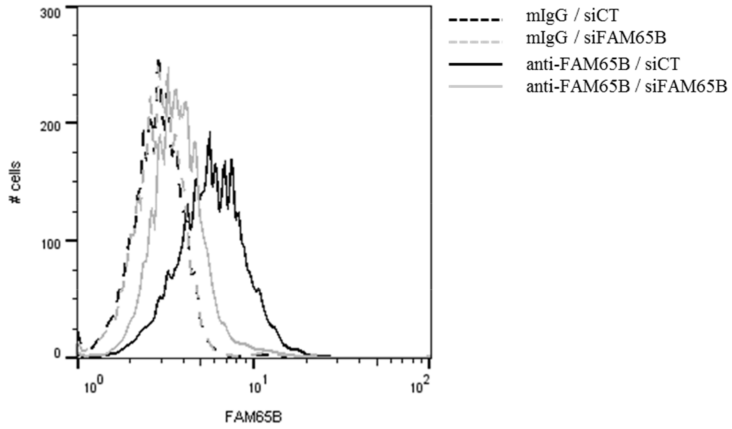

Supplementary Figure S5: Analyze of the expression of FAM65B after transfection of siRNA CT or FAM65B. Four days after transfection with siRNA CT or FAM65B, the human primary T cells were stained with anti-FAM65B antibodies or control immunoglobulin. These control experiments were systematically performed to check for extinction of FAM65B expression.

Supplementary Table S1: sequences of the primers use

		Séquence (5′ – > 3′)
peGFP-FAM65B RL151-152AA	F	tgtatccagcgaGCcGCccaggatggtgccagcaaaatgaagca
	R	tgcttcattttgctggcaccatcctggGCgGCtcgctggataca
peGFP-FAM65B S(x5)A	S21A_F	atcattagaagccagGcctttgcgggtttcagcggc
	S21A_R	gccgctgaaacccgcaaaggCctggcttctaatgat
	S37A_F	ggcgatccaggtgtaacGccttcattgaaaatt
	S37A_R	aattttcaatgaaggCgttacacctggatcgcc
	S523A _F	ctggtcaagaggctcacaGCTgcagaggtgccaatggcc
	S523A _R	ggccattggcacctctgcAGCtgtgagcctcttgaccag
	S535A _F	atggccacagacaggctgctcGCTgagggttctgttggtgga
	S535A _R	tccaccaacagaaccctcAGCgagcagcctgtctgtggccat
	S341A _F	cccttcagaggagaatgGCCatgtacagccagggtacc
	S341A _R	ggtaccctggctgtacatGGCcattctcctctgaaggg
pmCherry-FAM65B	F	atacatACCGGTCGCCACATGgtgagcaagggcgaggagga
	R	atgtatGCGGCCGCttacttgtacagctcgtccatgccg
pEF-FAM65B-myc	F	atacatACTAGTatgttggtaggatcccagtctttttcgcc
	R	gtcatgaatttggatgatattctaaaaaagGATATCatacat
ppia (qRT-PCR)	F	ggtgacttcacacgataatg
	R	acaagatgcaggacccgtat
Fam65b (qRT-PCR)	F	gcggagtttaacctcagcag
	R	ccttcaggtgtgactttggc
